# Iron Released after Cryo-Thermal Therapy Induced M1 Macrophage Polarization, Promoting the Differentiation of CD4^+^ T Cells into CTLs

**DOI:** 10.3390/ijms22137010

**Published:** 2021-06-29

**Authors:** Shicheng Wang, Man Cheng, Peng Peng, Yue Lou, Aili Zhang, Ping Liu

**Affiliations:** 1 School of Biomedical Engineering, Shanghai Jiao Tong University, Shanghai 200030, China; shichengwang@sjtu.edu.cn (S.W.); chengman@sjtu.edu.cn (M.C.); 112358answer@sjtu.edu.cn (P.P.); louyue4@163.com (Y.L.); zhangaili@sjtu.edu.cn (A.Z.); 2 School of Biomedical Engineering and Med-X Research Institute, Shanghai Jiao Tong University, Shanghai 200030, China

**Keywords:** cryo-thermal therapy, iron, M1 macrophages, CD4^+^ T cell differentiation, CD4 CTL

## Abstract

Macrophages play critical roles in both innate and adaptive immunity and are known for their high plasticity in response to various external signals. Macrophages are involved in regulating systematic iron homeostasis and they sequester iron by phagocytotic activity, which triggers M1 macrophage polarization and typically exerts antitumor effects. We previously developed a novel cryo-thermal therapy that can induce the mass release of tumor antigens and damage-associated molecular patterns (DAMPs), promoting M1 macrophage polarization. However, that study did not examine whether iron released after cryo-thermal therapy induced M1 macrophage polarization; this question still needed to be addressed. We hypothesized that cryo-thermal therapy would cause the release of a large quantity of iron to augment M1 macrophage polarization due to the disruption of tumor cells and blood vessels, which would further enhance antitumor immunity. In this study, we investigated iron released in primary tumors, the level of iron in splenic macrophages after cryo-thermal therapy and the effect of iron on macrophage polarization and CD4^+^ T cell differentiation in metastatic 4T1 murine mammary carcinoma. We found that a large amount of iron was released after cryo-thermal therapy and could be taken up by splenic macrophages, which further promoted M1 macrophage polarization by inhibiting ERK phosphorylation. Moreover, iron promoted DC maturation, which was possibly mediated by iron-induced M1 macrophages. In addition, iron-induced M1 macrophages and mature DCs promoted the differentiation of CD4^+^ T cells into the CD4 cytolytic T lymphocytes (CTL) subset and inhibited differentiation into Th2 and Th17 cells. This study explains the role of iron in cryo-thermal therapy-induced antitumor immunity from a new perspective.

## 1. Introduction

Macrophages play an important role in innate immune responses and are critical for priming and dictating T cell responses and differentiation [1]. Macrophages can be divided into two distinct subtypes, namely, classically activated proinflammatory macrophages (M1) and alternatively activated anti-inflammatory macrophages (M2) [2]. M1 macrophages mainly mediate proinflammatory processes that protect against tumors and play important roles in antitumor immunity, while M2 macrophages generally have anti-inflammatory, proangiogenic, metastasis-promoting and immunosuppressive functions [2]. The tumor microenvironment strongly polarizes macrophages toward an M2-like phenotype [3,4,5]. Therefore, reprogramming M2 macrophages to the M1 phenotype is a promising tumor therapeutic strategy [6,7].

In our previous study, we developed a cryo-thermal therapy technique that leads to complete regression of implanted tumors and extends long-term survival [8,9,10,11,12,13,14]. Cryo-thermal therapy has been shown to induce M1 macrophage polarization, leading to long-lasting, CD4^+^ T cell–mediated, Th1-dominant antitumor immunity [9,15,16]. HSP70 released after cryo-thermal therapy can promote M1 macrophage polarization [17]. However, it is also important to address whether other factors released in primary tumors after cryo-thermal therapy could induce M1 macrophage polarization.

Macrophages play a central role in the regulation of iron balance [18]. Aging red blood cells and excess iron can be recovered by macrophages to regulate the balance of iron [19,20]. Moreover, increasing evidence suggests that iron promotes M1 macrophage polarization [21,22,23,24,25]. Iron overload in macrophages induces an unrestrained proinflammatory phenotype, enhances TNF-α and hydroxyl radical release and impairs wound healing in humans and mice [23]. Iron accumulation in macrophages after spinal cord injury increases TNF expression and the emergence of a macrophage population with a proinflammatory mixed M1/M2 phenotype [22]. Indeed, lung adenocarcinoma patients with iron-positive tumors show better overall survival than those with iron-negative tumors [26].

The levels of iron and many proteins involved in iron metabolism have been found to be upregulated in cancer cells [27,28,29,30]. By using synchrotron radiation X-ray fluorescence imaging (SXRF, Shanghai Synchrotron Radiation Facility, Shanghai, China) and inductively coupled plasma mass spectrometry (ICP-MS, Element R., Thermo Fisher Scientific, Bremen, Germany), our previous study revealed that the iron content of 4T1 breast carcinoma tumor tissue gradually increases as the tumor grows [31]. Cryo-thermal therapy leads to tumor cell necrosis [32], which would result in the release of large amounts of iron. Moreover, an alternating cycle of cooling and heating after cryo-thermal therapy enhanced its effectiveness in damaging the tumor vasculature, leading to the release of red blood cells [33], which would also result in the release of iron. Hence, we hypothesized that cryo-thermal therapy could cause large amounts of iron to be released in primary tumors, leading to M1 macrophage polarization, which would further promote the activation of other immune cells and enhance systemic antitumor immunity.

In this study, we investigated iron released in primary tumors; the level of iron in splenic macrophages after cryo-thermal therapy; and the effect of iron on macrophage polarization, dendritic cell (DC) maturation and CD4^+^ T cell differentiation in metastatic 4T1 murine mammary carcinoma. We found that a large quantity of iron was released from the tumor site after cryo-thermal therapy; this iron was taken up by splenic macrophages and promoted M1 macrophage polarization by inhibiting ERK phosphorylation. Moreover, the released iron promoted the maturation of DCs after cryo-thermal therapy. Finally, iron-induced M1 macrophages and DC maturation after cryo-thermal therapy promoted the differentiation of CD4^+^ T cells into CD4 CTLs and inhibited differentiation into Th2 and Th17 cells. Our findings further explained the mechanism of cryo-thermal therapy-induced antitumor immunity from the perspective of trace elements.

## 2. Results

### 2.1. Cryo-Thermal Therapy Induces Tumor Vessel Rupture, Cell Disruption and Iron Release in Primary Tumors

**Figure 1:**
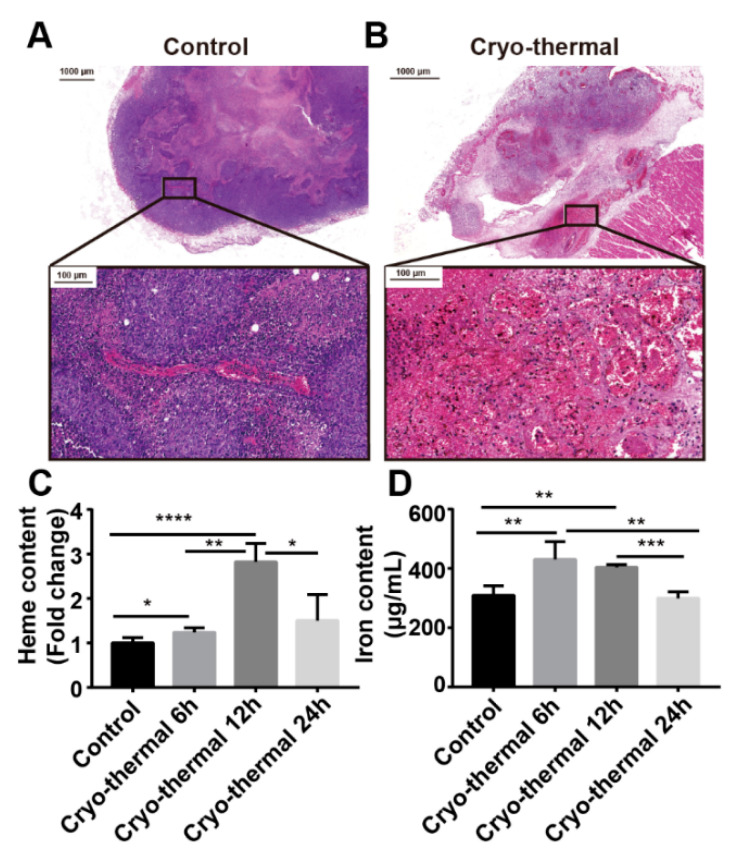
Iron released from tumor after cryo-thermal therapy. (**A**,**B**) Representative H&E staining of the tumors before and after cryo-thermal therapy. (**C**) Heme levels in tumor interstitial fluid 6 h, 12 h and 24 h after cryo-thermal therapy. (**D**) Iron quantification in tumor interstitial fluid 6 h, 12 h and 24 h after cryo-thermal therapy. * *p* < 0.05, ** *p* < 0.01, *** *p* < 0.001, **** *p* < 0.0001, *n* = 3 for each group.

Our previous study has shown that cryo-thermal therapy via pre-cooling before heating induces massive destruction of tumor blood vessels [33] and tumor cell necrosis in tumor tissues [8]. We hypothesized that necrosis of tumor cells and rupture of blood vessels would lead to the release of large amounts of iron after cryo-thermal therapy. Necrosis of tumor tissue and destruction of vessels were evaluated by Hematoxylin & Eosin (H&E) staining. At 24 h after cryo-thermal therapy, tumor cell plasma membranes were found to be ruptured and numerous red blood cells were dispersed throughout the tumor tissues, indicating that tumor cells and blood vessels were destroyed (Figure 1B). Iron content and heme levels in tumor interstitial fluid were measured by using ICP-MS after cryo-thermal therapy. The level of heme in tumor interstitial fluid was increased at 6 h and reached a peak at 12 h after cryo-thermal therapy (Figure 1C). The iron content in tumor interstitial fluid was also increased at 6 h and 12 h after cryo-thermal therapy (Figure 1D). These results indicated that iron was released and accumulated at the tumor site from 6 h to 24 h after cryo-thermal therapy.

### 2.2. Iron Released from the Primary Tumor after Cryo-Thermal Therapy Was Taken Up by Splenic Macrophages

**Figure 2:**
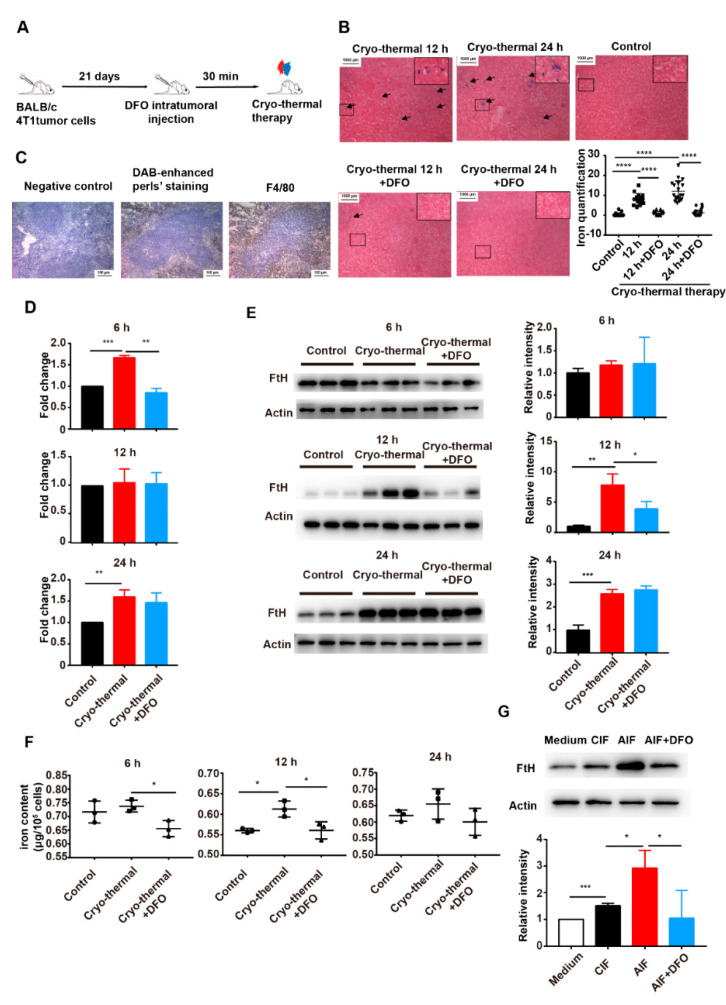
Splenic macrophages taken up the iron after cryo-thermal therapy. (**A**) Schematic of experimental design. 4T1 tumor-bearing mice were injected with DFO introtumoral 30 min before cryo-thermal therapy and spleens were harvested for analyzing the changes in iron. (**B**) Representative Perls’ staining of the spleen after cryo-thermal therapy with or without DFO injection. (**C**) Representative DAB-enhanced Perls’ staining (middle) and immunohistochemistry staining using an F4/80 antibody (right). (**D**,**E**) Splenic macrophages were sorted and the mRNA levels and protein levels (fold change) of FtH were determined by RT-qPCR and western blot, respectively. (**F**) F4/80^+^ macrophages were sorted by flow cytometer and iron contents were quantified by ICP-MS. (**G**) RAW264.7 cells were treated with tumor interstitial fluid from control or cryo-thermal treated mice. DFO was added to chelate iron in the medium. After 24 h of cultivation, protein levels of FtH were determined by western blot. * *p* < 0.05, ** *p* < 0.01, *** *p* < 0.001, **** *p* < 0.0001, *n* = 3 for each group.

As blood flows through the spleen, aging red blood cells and iron are engulfed, processed and stored by spleen macrophages to complete the process of iron recovery [34]. As iron was released from 6 h to 24 h after cryo-thermal therapy, we evaluated the accumulation of iron in the spleen by Perls’ staining at 12 h and 24 h after treatment. Positive staining for iron was detected in spleens from cryo-thermal treated mice but not in spleens from untreated mice (Figure 2A). Quantification of iron staining showed that the spleens of cryo-thermal treated mice had significantly higher iron content at 12 h and 24 h after treatment than the spleens of the control group (tumor-bearing mice) (Figure 2B). In order to further investigate whether the increased iron content came from cryo-thermal therapy-treated tumors, deferoxamine (DFO), an iron chelator, was injected intratumorally 30 min before cryo-thermal therapy. With DFO injection, the positive Perls’ staining in the spleen disappeared (Figure 2B). These results suggested that cryo-thermal therapy could induce iron accumulation in the spleen.

Macrophages in the spleen serve the function of iron recovery [34] and we investigated whether iron would accumulate in macrophages after cryo-thermal therapy. Two consecutive frozen slices were used. One slice was stained by DAB-enhanced Perls’ staining (Figure 2C, middle) to visualize iron [35] In addition, in another slice, macrophages were immunolabeled with anti-F4/80 and stained with HRP labeled secondary antibody and DAB reagent (Figure 2C, right). The immunohistochemical results showed that there was obvious overlap in the stained positive area of the two slices and positive staining for F4/80, a widely used tissue macrophage marker in mice, was colocalized with positive DAB-enhanced Perls’ staining positive region, which suggested that iron was taken up by splenic macrophages (Figure 2C).

Iron in macrophages is mainly stored in ferritin [36]. The expression level of ferritin reflects the level of iron in cells [37]. In order to further explore the absorption of iron by macrophages, splenic macrophages were sorted by magnetic separation and the expression level of ferritin in macrophages was investigated by using RT-qPCR and western blot. After cryo-thermal therapy, the mRNA level of ferritin heavy chain (FtH) was upregulated in spleen macrophages at 6 h and 12 h (Figure 2D). Additionally, the protein level of FtH was increased at 12 h after cryo-thermal therapy (Figure 2E). However, the upregulation of FtH mRNA and protein levels at 6 h and 12 h after cryo-thermal therapy was eliminated by DFO treatment (Figure 2D,E). The upregulation of the iron storage protein FtH indicated that the iron content of macrophages was increased after cryo-thermal therapy.

Splenic F4/80^+^ macrophages were sorted by using flow cytometry and the iron content of the macrophages was determined by using ICP-MS. After cryo-thermal therapy, the iron content of splenic macrophages was markedly increased at 12 h and slightly increased at 24 h in the treated mice compared to the control group, but splenic macrophage iron levels were decreased at 6 h and 12 h after DFO treatment (Figure 2F).

We further explored the absorption of iron in macrophages in vitro. Tumor interstitial fluid was harvested 6 h after cryo-thermal therapy (AIF). The RAW264.7 mouse macrophage cell line was cultured with 2% AIF or interstitial fluid from tumor-bearing mice (CIF). The expression level of FtH in macrophages was significantly upregulated in culture with AIF compared to culture with CIF (Figure 2G). However, the upregulation of FtH expression was abrogated after DFO treatment (Figure 2G). Taken together, these findings suggested that the iron released from the primary tumor after cryo-thermal therapy was taken up by macrophages.

### 2.3. The Iron Released from the Primary Tumor Promoted M1 Polarization after Cryo-Thermal Therapy

**Figure 3:**
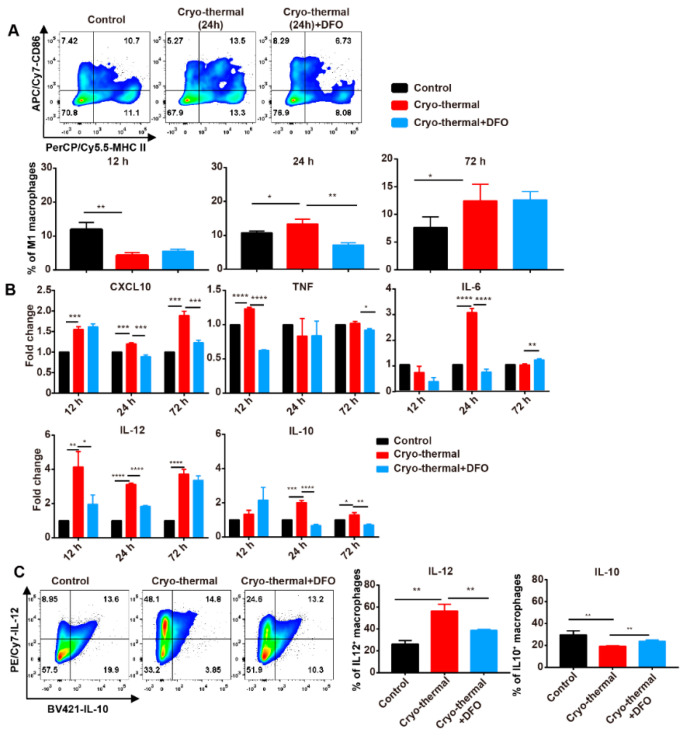
DFO treatment inhibited the M1 polarization of macrophages. Mice were sacrificed and spleen were harvested 12 h, 24 h and 72 h after cryo-thermal therapy. (**A**) The percentages of M1 macrophages (CD86^+^MHC II^+^) were measured by using flow cytometry. (**B**) Macrophages were sorted and the expression levels of *CXCL10*, *TNF*, *IL-6*, *IL-12* and *IL-10* were determined by using RT-qPCR. (**C**) Intracellular IL-12 and IL-10 in macrophages were determined by using flow cytometry. * *p* < 0.05, ** *p* < 0.01, *** *p* < 0.001, **** *p* < 0.0001, *n* = 3 for each group.

In order to investigate the impact of iron on M1 polarization of macrophages after cryo-thermal therapy, mice were treated by cryo-thermal therapy alone or cryo-thermal therapy combined with intratumoral injection of DFO. The frequency of M1macrophages (CD86^+^ MHC II^+^) was detected by flow cytometry (Figure S1). As in our previous results, the percentage of M1 macrophages in the spleen was reduced at 12 h but increased at 24 h and 72 h after cryo-thermal therapy (Figure 3A). However, the M1 macrophage polarization induced by cryo-thermal therapy was abrogated at 24 h with DFO treatment (Figure 3A), which indicated that cryo-thermal therapy could promote M1 macrophage polarization and that the release of iron in primary tumors was indispensable for M1 macrophage polarization induced by cryo-thermal therapy. M1 macrophages are characterized by high expression of the proinflammatory cytokines TNF-α, IL-6 and IL-12 and the chemokine CXCL10 [1]. In order to explore the effect of the released iron on the function of macrophages after cryo-thermal therapy, the expression levels of *TNF-α*, *IL-6*, *IL-12*, *IL-10* and *CXCL10* were quantified by using quantitative reverse transcription PCR (RT-qPCR). The mRNA levels of *TNF-α* and *IL-6* were upregulated at 12 h and 24 h after cryo-thermal therapy and the expression levels of *IL-12* and the chemokine *CXCL10* were increased from 6 h to 72 h after cryo-thermal therapy compared with those of the control group. However, DFO treatment blunted the upregulation of *TNF-α*, *IL-6*, *CXCL10* and *IL-12* induced by cryo-thermal therapy (Figure 3B). *IL-10* is a major immunosuppressive cytokine [38]. The mRNA expression level of *IL-10* was upregulated after cryo-thermal therapy and downregulated with DFO treatment, but the level of *IL-10* was much lower than that of *IL-12* (Figure 3B). Meanwhile, the levels of IL-12 and IL-10 in macrophages were measured by using flow cytometry. Compared with the control group, high levels of IL-12 and low levels of IL-10 in splenic macrophages were observed in the cryo-thermal therapy group; however, DFO treatment inhibited the increase in IL-12 and the decrease in IL-10 induced by cryo-thermal therapy (Figure 3C). These results indicated that the iron released in primary tumors after cryo-thermal therapy could promote M1 polarization of macrophages, characterized by the upregulation of MHC II, CD86, proinflammatory cytokines and chemokines.

### 2.4. The Iron Released from the Primary Tumor Promoted M1 Polarization by Inhibiting the Phosphorylation of ERK after Cryo-Thermal Therapy

**Figure 4:**
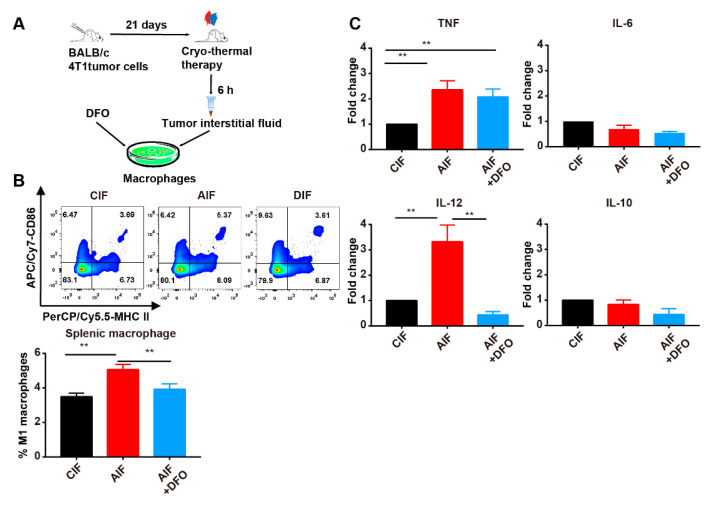
Iron promoted M1 polarization of macrophages in vitro. (**A**) Schematic of experimental design. Tumor interstitial fluid was havested from tumor-bearing mice (CIF) or treated mice (AIF). Splenic macropahges sorted from tumor-bearing mice were treated with AIF, CIF or combined with DFO treatment. (**B**) The percentages of M1 macrophages (CD86^+^MHC II^+^) were measured by flow cytometry and (**C**) the expression levels of *TNF*, *IL-6*, *IL-12* and *IL-10* were determined by RT-qPCR. ** *p* < 0.01, *n* = 3 for each group.

We next explored the direct effect of the released iron on the M1 polarization of macrophages in vitro. Splenic macrophages were cultured with AIF or CIF in the presence or absence of DFO for 24 h and the phenotype and function of macrophages were detected by using flow cytometry and RT-qPCR. AIF treatment increased the percentage of M1 macrophages compared to CIF treatment, while AIF with DFO treatment blunted the increase in the percentage of M1 macrophages (Figure 4B). In AIF-treated cells, compared with CIF-treated cells, the expression levels of *IL-6* and *IL-10* were not obviously changed; however, the expression levels of *TNF* and *IL-12* were increased significantly. AIF with DFO treatment had no effect on the expression of *IL-6*, *IL-10* or *TNF* but dramatically decreased the expression level of *IL-12* (Figure 4C). These results were further validated using two other macrophage populations, peritoneal macrophages and RAW264.7 cells. The percentage of M1 macrophages (CD86^+^ MHC II^+^) was not changed after culture with AIF, but it was decreased after culture with AIF and DFO compared to culture with AIF alone (Figure S2A). Similarly, the expression level of *IL-10* was not changed after culture with AIF and decreased slightly after culture with AIF and DFO. However, AIF treatment significantly increased the expression levels of *TNF*, *IL-6* and *IL-12*, whereas AIF with DFO treatment downregulated the expression of proinflammatory cytokines in peritoneal macrophages and RAW264.7 cells (Figure S2B,C). Taken together, these results suggested that iron released from primary tumors promoted the M1 polarization of macrophages after cryo-thermal therapy.

**Figure 5:**
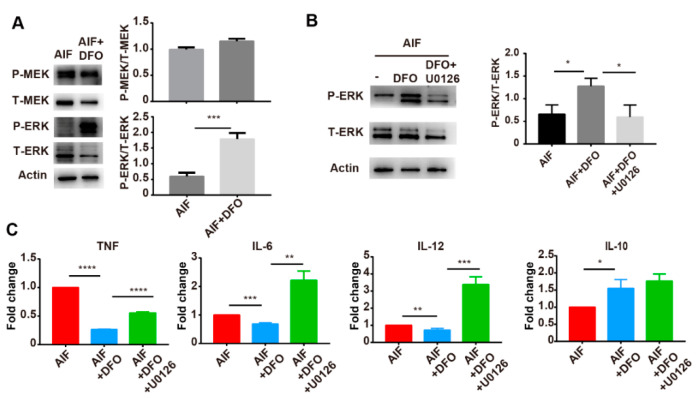
Iron promoted M1 polarization of macrophages by inhibiting the phosphorylation of ERK. (**A**) RAW264.7 cells were treated with AIF with or without DFO treatment for 24 h, the MEK/ERK pathway was analyzed by western blot. (**B**) Cells were pretreated with U0126, an inhibitor of MEK/ERK pathway, for 30 min to inhibit the phosphorylation of ERK. The phosphorylation of ERK was analyzed by Western blot and (**C**) the expression levels of *TNF*, *IL-6*, *IL-12* and *IL-10* were measured by Real-Time qPCR. Experiments were performed in duplicate. * *p* < 0.05, ** *p* < 0.01, *** *p* < 0.001, **** *p* < 0.0001.

The ERK signaling pathway can be activated by an iron chelator [39]. Activation of the ERK signaling pathway promotes M2 polarization of macrophages and inhibits IL-12 expression [40,41,42,43]. Therefore, we measured the phosphorylation of MEK/ERK in RAW264.7 cells cultured with AIF or AIF with DFO treatment for 24 h. After AIF with DFO treatment, the phosphorylation level of MEK was not changed, but the phosphorylation level of ERK was significantly increased compared to AIF treatment alone (Figure 5A). In order to investigate the relationship between ERK phosphorylation and macrophage polarization after combined AIF and DFO treatment, RAW264.7 cells were pretreated for 30 min with U0126, an ERK inhibitor and then cultured with AIF and DFO. The expression of M1 macrophage-associated cytokines was measured by using RT-qPCR. The phosphorylation of ERK after AIF with DFO treatment was inhibited by U0126 treatment (Figure 5B). As in the previous results, AIF with DFO treatment downregulated the expression of *TNF*, *IL-6* and *IL-12*, but the expression of these genes was increased after pretreatment with U0126 compared with AIF. With DFO treatment, the levels of *IL-6* and *IL-12* were even higher than those in the AIF group (Figure 5C). Altogether, these results suggested that the iron released from the primary tumor after cryo-thermal therapy promoted M1 polarization of macrophages by inhibiting the phosphorylation of ERK.

### 2.5. Iron Participated in the Functional Maturation of DCs after Cryo-Thermal Therapy

**Figure 6:**
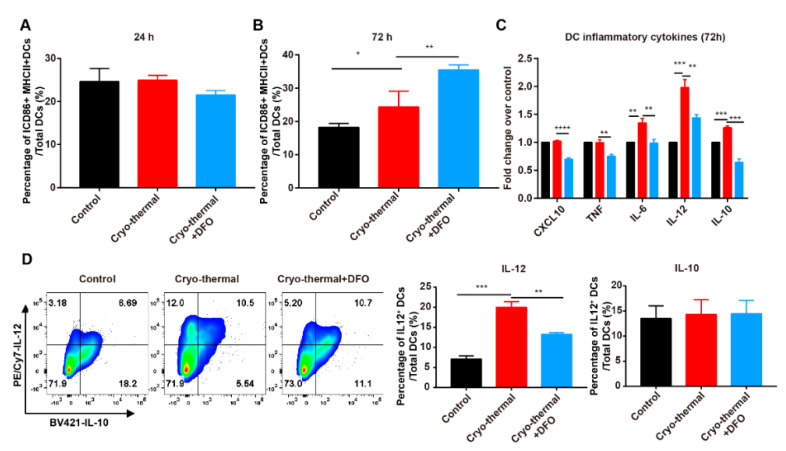
Iron promotes DCs maturation. Mice were sacrificed and spleens were harvested 24 h and 72 h after cryo-thermal therapy. (**A**,**B**) The percentages of mature DCs (CD86^+^MHC II^+^) were measured by flow cytometry. (**C**) DCs were sorted and the expression levels of *CXCL10, TNF, IL-6, IL-12* and *IL-10* were determined by RT-qPCR. (**D**) Intracellular IL-12 and IL-10 in DCs were determined by flow cytometry. * *p* < 0.05, ** *p* < 0.01, *** *p* < 0.001, **** *p* < 0.0001, *n* = 3 for each group.

Our previous studies showed that cryo-thermal therapy modulates the phenotypic and functional maturation of splenic DCs [16,44]. Based on these results, the markers of mature CD11c^+^ DCs were determined by using flow cytometry (Figure S1) and RT-qPCR. At 24 h after cryo-thermal therapy, the percentage of CD86^+^ MHC II^+^ DCs in the spleen was not obviously different after 24 h but was increased after 72 h compared to the control group (Figure 6A,B). DFO treatment did not affect the expression of CD86 and MHC II on DCs at 24 h but increased the expression of CD86 and MHC II after cryo-thermal therapy (Figure 6A,B). The mRNA levels of *IL-6*, *IL-12* and *IL-10* in DCs were increased after cryo-thermal therapy and the increased level of IL-12 was most significant. Interestingly, the mRNA levels of *CXCL10*, *TNF-α*, *IL-6*, *IL-12* and *IL-10* were markedly decreased after cryo-thermal therapy with DFO treatment (Figure 6C). The protein levels of IL-12 and IL-10 were also determined by using flow cytometry. Similarly, the expression of IL-12 in DCs was increased after cryo-thermal therapy, but the increase in IL-12 was abolished after DFO treatment (Figure 6D). These results indicated that iron also participated in the functional maturation of DCs after cryo-thermal therapy.

### 2.6. Iron-Induced M1 Macrophages and Mature DCs Promoted the Differentiation of CD4^+^ T Cells into the CD4 CTL Subset after Cryo-Thermal Therapy

**Figure 7:**
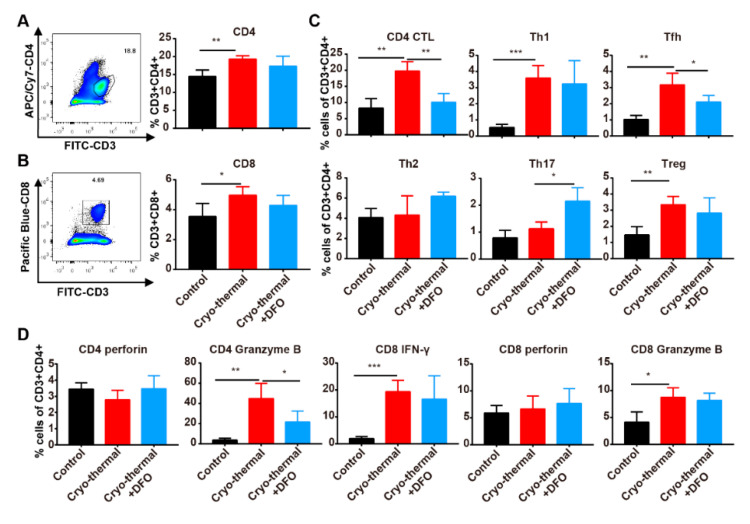
Iron promoted the differentiation of CD4 CTL and Tfh. Mice were sacrificed and spleens were harvested 72 h after cryo-thermal therapy. (**A**,**B**) The percentages of CD4^+^ and CD8^+^ T cells were measured by flow cytometry. (**C**) CD4 CTL (Thpok^-^), Th1 (IFN-γ^+^), Tfh (Bcl6^+^), Th2 (IL-4^+^), Th17 (IL-17^+^) and Treg (Foxp3^+^) subsets in CD4^+^ T cells were measured by flow cytometry. (**D**) Intracellular granzyme B, perforin and IFN-γ^+^ in CD4^+^ and CD8^+^ T cells were determined by flow cytometry. * *p* < 0.05, ** *p* < 0.01, *** *p* < 0.001, *n* = 4 for each group.

The maturation of innate immune cells can further activate adaptive immune cells; therefore, we next investigated the effect of iron on the differentiation of T cells after cryo-thermal therapy. A significant increase in the abundance of CD4^+^ T cells and CD8^+^ T cells in the spleen was found on day 3 after cryo-thermal therapy, but there was no difference compared to cryo-thermal therapy combined with DFO treatment (Figure 7A,B). Then, the differentiation of CD4^+^ T cells was analyzed by using flow cytometry. As in our previous studies, the percentages of CD4 CTLs, Th1 cells, Tfh cells and regulatory T cells (Tregs) were markedly increased after cryo-thermal therapy compared to the control group, but the levels of CD4 CTLs, Th1 cells and Tfh cells were much higher than the level of Tregs (Figure 7C and Figure S3). The percentages of Th2 and Th17 cells were not obviously changed after cryo-thermal therapy (Figure 7C and Figure S3). However, the increased percentages of CD4 CTLs and Tfh cells induced by cryo-thermal therapy were abolished by DFO treatment. After cryo-thermal therapy with DFO treatment, the percentages of CD4 CTLs and Tfh cells were decreased and there was an upward trend in the percentage of Th2 cells compared to cryo-thermal therapy alone (Figure 7C and Figure S3). Interestingly, cryo-thermal therapy with DFO treatment upregulated the percentage of Th17 cells, which reportedly play both antitumorigenic and protumorigenic roles [45]. The expression of cytotoxic cytokines in T cells was also determined by using flow cytometry. Compared to the control group, the expression of granzyme B in CD4^+^ T cells was obviously upregulated in the cryo-thermal therapy group but was decreased in the combined cryo-thermal therapy and DFO treatment (Figure 7D and Figure S4A). The expression of perforin in CD4^+^ and CD8^+^ T cells was not affected after cryo-thermal therapy with DFO treatment (Figure 7D and Figure S4). The expression of granzyme B and IFN-γ in CD8^+^ T cells was increased after cryo-thermal therapy, but there was no significant difference compared to cryo-thermal therapy with DFO treatment (Figure 7D and Figure S4B). These results indicated that iron promoted the differentiation of CD4^+^ T cells to CTLs and Tfh cells, inhibited the differentiation of CD4^+^ T cells to Th2 and Th17 cells and enhanced the cytotoxicity of CD4^+^ T cells after cryo-thermal therapy.

**Figure 8:**
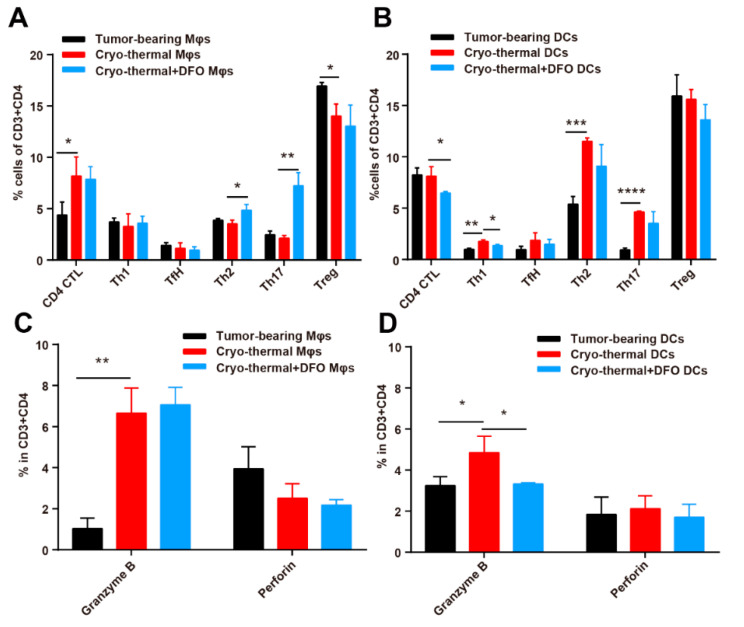
Iron-induced macrophages and DCs after cryo-thermal therapy promoted the differentiation of CD4 CTL. Mice were sacrificed and spleens were harvested 72 h after cryo-thermal therapy. Macrophages and DCs were sorted from tumor-bearing mice, cryo-thermal group and DFO treated cryo-thermal group mice and cocultured with CD4^+^ T cells for 24 h. (**A**,**B**) CD4 CTL (Thpok^−^), Th1 (IFN-γ^+^), Tfh (Bcl6^+^), Th2 (IL-4^+^), Th17 (IL-17^+^) and Treg (Foxp3^+^) subsets in CD4^+^ T cells were measured by using flow cytometry. (**C**,**D**) Intracellular granzyme B and perforin in CD4^+^ T cells were determined by flow cytometry. * *p* < 0.05, ** *p* < 0.01, *** *p* < 0.001, **** *p* < 0.0001, *n* = 3 for each group.

Our previous studies documented that cryo-thermal-induced M1 macrophages were required for the promotion of polyfunctional CD4^+^ T cells [16]. Here, we aimed to explore whether iron-induced M1 macrophages and mature DCs after cryo-thermal therapy could affect the differentiation of CD4^+^ T cells. Splenic CD68^+^ macrophages and CD11c^+^ DCs from tumor-bearing mice (tumor-bearing Mφs or DCs), cryo-thermal group (cryo-thermal Mφs or DCs) and DFO treated cryo-thermal group (cryo-thermal + DFO Mφs or DCs) were sorted by using magnetic beads and cocultured for 24 h with CD4^+^ T cells derived from tumor-bearing mice 24 days after inoculation. The differentiation of CD4^+^ T cells was assessed by using flow cytometry. Compared with tumor-bearing Mφs, cryo-thermal Mφs promoted the differentiation of CD4^+^ T cells to CD4 CTLs and reduced the percentage of Tregs, while the percentages of Th1, Tfh, Th2 and Th17 cells were not changed (Figure 8A and Figure S5A). However, Mφs from cryo-thermal therapy with DFO treatment increased the percentages of the Th2 and Th17 subsets compared to cryo-thermal Mφs (Figure 8A and Figure S5A). Compared with tumor-bearing DCs, cryo-thermal DCs increased the percentage of Th1, Th2 and Th17 subsets but did not affect the differentiation of CD4 CTL, Tfh, or Tregs (Figure 8B and Figure S5B). DCs from cryo-thermal therapy with DFO treatment downregulated the percentage of CD4 CTL and Th1 as compared to cryo-thermal DCs (Figure 8B and Figure S5B). Both cryo-thermal Mφs and cryo-thermal DCs increased the level of granzyme B in CD4^+^ T cells compared to the tumor-bearing group, but DCs from cryo-thermal therapy with DFO treatment decreased the level of granzyme B in CD4^+^ T cells (Figure 8C,D and Figure S6). The expression of perforin in CD4^+^ T cells was not markedly change in these groups. These in vitro results suggested that iron-induced M1 macrophages and mature DCs promoted the differentiation of CD4^+^ T cells into the CD4 Th1 and CTL subsets and inhibited the differentiation of CD4^+^ T cells into Th2 and Th17 subsets, which was consistent with the in vivo results.

## 3. Discussion

Our previous studies demonstrate that cryo-thermal therapy can not only effectively ablate tumors locally but also induce systematic antitumor immunity [8,9,10,15,17,44,46,47]. After cryo-thermal therapy, tumors in situ release tumor antigens and danger signals, which facilitate a durable antitumor adaptive immune response [15,17,47]. However, the existing research had not yet addressed whether other components released from tumors in situ after cryo-thermal therapy trigger host immunity. In this study, we found that cryo-thermal therapy induced tumor cell necrosis and vascular destruction, which led to the massive release of iron and iron-containing heme. The released iron was taken up by splenic macrophages and promoted M1 macrophage polarization, characterized by high expression of MHC II, CD86 and proinflammatory cytokines. At the same time, this iron also promoted the functional maturation of DCs. Finally, the iron-induced M1 macrophages and mature DCs promoted the differentiation of CD4^+^ T cells into CTLs, enhancing the cytotoxicity of CD4^+^ T cells and inhibited the differentiation of CD4^+^ T cells into Th2 and Th17 cells.

After cryo-thermal therapy, tumor cells and tumor blood vessels were largely destroyed, resulting in increased levels of iron and heme in tumors in situ. The spleen is a lymphoid organ that filters the blood. It removes old red blood cells and recycles iron. When blood flows through the spleen, senescent red blood cells, free heme and excess iron can be engulfed and processed by splenic macrophages for iron recovery [34]. In this study, iron content and FtH levels were increased in splenic macrophages, indicating that the released iron was taken up by splenic macrophages. Cryo-thermal therapy induces macrophage polarization to the M1 phenotype [15,16]. Iron can promote M1 macrophage polarization [22,24,25,35,48]. In this study, we revealed that, after cryo-thermal therapy, the released iron promoted M1 macrophage polarization, which could be attributed to iron inhibiting ERK phosphorylation.

It is reported that DFO can chelate extracellular iron and inhibit the expression of proinflammatory cytokines in macrophages [21,48,49,50]. Moreover, an intracellular iron chelator (TC3-S)_2_ can target intracellular iron and shift the macrophage phenotype from iron release towards sequestration, a feature of M1 macrophages [51]. In this study, we hypothesized that cryo-thermal therapy could cause large amounts of iron to be released in primary tumor leading to M1 macrophage polarization. To investigate the effect of the released iron on macrophage polarization after cryo-thermal therapy, we chose the extracellular iron chelator DFO to chelate the released iron after cryo-thermal therapy.

It is generally believed that macrophages are involved in iron metabolism and that macrophages express a range of factors mediating iron uptake and recycling [34]. However, there have been no detailed studies describing the role of DCs in systemic iron metabolism. In the present study, we found that iron could promote the functional maturation of DCs. Considering that cryo-thermal therapy-induced M1 macrophages reshape immature DCs to fully mature DCs [15,16], we cocultured macrophages with DCs. Macrophages from mice that received combined cryo-thermal and DFO treatment impaired DC maturation, which was characterized by decreased expression of CD86 and MHC II in DCs (Figure S7). We suggested that the effect of iron on DC maturation is an indirect effect mediated by macrophages. However, the mechanism needs to be further explored.

CD4^+^ T cells play a pivotal role in eliciting vigorous antitumor immune responses [52]. CD4 CTLs are a CD4 subset with cytotoxic activity and can directly kill tumor cells by secreting granzyme B and perforin, while Th2 and Th17 cells inhibit cell-mediated immunity and promote tumor progression [53,54,55,56]. Our in vivo results showed that iron promoted the differentiation of CD4 CTL and inhibited the differentiation of Th2 and Th17 cells after cryo-thermal therapy, implying the role of iron in antitumor immunity after cryo-thermal therapy. Iron is involved in the survival, proliferation and development of T cells [57], but no study has shown that iron can regulate the differentiation of CD4^+^ T cells. Our previous study has demonstrated that macrophages and DCs promote the differentiation of CD4^+^ T cells into cytotoxic Th1 and CTL cells [16]. Given that iron promoted M1 macrophage polarization and DC maturation, we cocultured macrophages and DCs with CD4^+^ T cells. We found that after cryo-thermal therapy, M1 macrophages inhibited the differentiation of CD4^+^ T cells to Th2 and Th17 cells and mature DCs promoted the differentiation of CD4^+^ T cells to CTLs depending on iron release induced by cryo-thermal therapy.

The expression of CD86 and MHCII in macrophages represents phenotypic polarization of macrophages. Peritoneal macrophages induced by Thioglycollate Broth were biased towards phenotypic polarization of M1 macrophages with high expression levels of CD86 and MHCII (Figure S2A) and AIF could not further increase the expression of CD86 and MHCIIin peritoneal macrophages, so this effect that AIF promoted phenotypic polarization of M1 macrophages was not observed in peritoneal macrophages. However, RT-qPCR results showed that compared with CIF treatment, AIF treatment increased the expression of proinflammatory cytokines characterized by functional polarization of M1 macrophages in splenic macrophages, peritoneal macrophages and RAW264.7 macrophage cell line (Figure 3B and Figure S2B,C), which indicated that, though AIF could not further promote phenotypic polarization of M1 macrophages in peritoneal macrophages, it could promote functional polarization of M1 macrophages. Thus, the effect of AIF on phenotypic polarization of macrophages was not dependent on the origin of macrophage, but was dependent on the polarization state of macrophages. Importantly, AIF could promote functional polarization of M1 macrophages.

Although the percentage of M1 phenotypic polarization of macrophages (CD86^+^MHC II^+^) in control mice was higher than that at 12 h after cryo-thermal therapy, but its expression of proinflammatory cytokines in macrophages characterized by functional polarization of M1 macrophages were decreased much lower than that at 12 h after cryo-thermal therapy, which indicated that macrophages from control mice were not fully M1-polarization macrophages. At 12 h after cryo-thermal therapy, the expression of M1 surface marker CD86^+^MHC II^+^ in macrophages was decreased, but the expression of IL-12 in macrophages was significantly increased compared with that in control group, indicating that cryo-thermal therapy significantly promoted functional polarization of M1 macrophages at 12 h after cryo-thermal therapy. The decrease of M1 macrophages 12 h after cryo-thermal therapy would be caused by the cryo-thermal-induced acute inflammatory environment, but the mechanism needs to be further explored.

In summary, our study showed that iron released after cryo-thermal therapy promoted M1 macrophage polarization and DC maturation and iron-induced M1 macrophages and mature DCs promoted subsequent CD4^+^ T-cell differentiation into CD4 CTLs and inhibited differentiation into Th2 and Th17 cells. This study suggested that cryo-thermal therapy could induce the release of iron to effectively activate macrophages, which would further explain the mechanism of cryo-thermal therapy-induced antitumor immunity from the perspective of trace elements.

## 4. Materials and Methods

### 4.1. Animal Model

Female Balb/C mice were obtained from Shanghai Slaccas Experimental Animal Co., Ltd. (Shanghai, China). They were housed and fed sterile food with standard mice nutritional formula and sterile water in the isolated cages of 12 h light/dark cycle environment. All animal experiments were approved by the Animal Welfare Committee of Shanghai Jiao Tong University and experimental methods were performed in accordance with the guidelines of Shanghai Jiao Tong University Animal Care (approved by Shanghai Jiao Tong University Scientific Ethics Committee, Proto code 2020017, 26 March 2020). 4T1 cells (provided by Shanghai First People’s Hospital, Shanghai, China) were cultured in DEME medium (Hyclone, Logan, UT, USA) supplemented with 10% fetal bovine serum (FBS, Gemini Bio-Products, West Sacramento, CA, USA) and penicillin-streptomycin (Hyclone, Logan, UT, USA). To prepare the tumor-bearing mice, 4T1 cells (5 × 10^5^) were injected subcutaneously into right femoral region of mice when the mice were 6–8 weeks old and weighed 20 g.

### 4.2. The Cryo-Thermal Therapy Procedures

Three weeks after tumor inoculation when the average tumor size reached about 0.2 cm^3^, mice were divided into three groups: tumor-bearing group without any treatment, cryo-thermal group and iron chelation group. The cryo-thermal therapy was performed as described previously [8,16]. Briefly, the subcutaneous tumor of mice in cryo-thermal therapy group was frozen with liquid nitrogen, then rewarming at room temperature and heated with radiofrequency (RF). To chelate the iron released after cryo-thermal therapy, mice were pre-injected with 10 mg deferoxamine mesylate salt (DFO, Sigma-Aldrich, St. Louis, MO, USA) intratumorally and subsequently treated with cryo-thermal therapy.

### 4.3. Preparation of Tumor Interstitial Fluid

Tumors were harvested one day after treatments. Tumor tissue was wrapped with Nylon filter paper with a pore size of 50 μm and centrifuged at 2000 rpm in a 15 mL centrifuge tube for 20 min. Tumor interstitial fluid was collected.

### 4.4. Inductively Coupled Plasma-Mass Spectrometry (ICP-MS) Analysis

To prepare splenic F4/80^+^ macrophages, spleens were grinded using GentleMACS™ dissociator (Miltenyi Biotec, Bergisch Gladbach, Germany) and red blood cells were removed by erythrocyte-lysing reagent containing 0.15 M NH_4_Cl, 1.0 M KHCO_3_ and 0.1 mM Na2EDTA. Splenic F4/80+ macrophages were sorted by FACS Aria II cytometer (BD Biosciences, San Jose, CA, USA). Splenic F4/80^+^ macrophages and tumor interstitial fluid were digested in 70% trace metal basis nitric acid (Sigma-Aldrich, St Louis, MO, USA) and then diluted with ddH_2_O. The iron contents of the samples were determined by ICP-MS (Element R., Thermo Fisher Scientific, Bremen, Germany).

### 4.5. Heme Content Determination

For the determination of the heme levels, 1 μL of tumor interstitial fluid was mixed with 200 μL of 2 M oxalic acid (Sigma-Aldrich, St Louis, MO, USA) and the solution was heated to 95 °C for 30 min. The samples were then centrifuged for 10 min at 1000× *g* at 4 °C to remove debris. The supernatant was moved to an opaque 96-well culture plate and the fluorescence was assessed at 405/600 nm by using a Spectra Max Gemini fluorescence microplate reader.

### 4.6. Flow Cytometry Analysis

The spleens were collected after therapy. Single-cell suspension of splenocytes was prepared using GentleMACS™ dissociator (Miltenyi Biotec, Bergisch Gladbach, Germany). Red blood cells were removed by erythrocyte-lysing reagent containing 0.15 M NH4Cl, 1.0 M KHCO3 and 0.1 mM Na2EDTA. Staining antibodies including CD11b-Pacific blue (clone M1/70), F4/80-APC (clone BM8), CD86-APC/cy7 (clone GL-1), CD86-PE (clone GL-1), IA-IE-percp-cy5.5 (clone M5/114.15.2), CD11c-PE (clone N418), CD3-FITC (clone 145-2C11), CD4-APC/Cy7 (clone RM4.5), CD8-Pacific blue (clone 53-6.7), IL-4-BV421 (clone 11B11), IL-17-PE (clone TC11-18H10.1), Perforin-PE (clone S16001B), Granzyme-B-AF647 (clone GB11), Bcl-6-percp-cy5.5 (clone 7D1), PD-1-PE/cy7 (clone 29F.1A12) and Foxp3-PE (clone MF-14) were purchased from Biolegend (San Diego, CA, USA). ThPok-AF647 (clone T43-94) and IFN-γ-Texas Red (clone XMG1.2) were purchased from BD Bioscience (San Jose, CA, USA). For cell surface staining, splenocytes were stained with antibodies as described above, for 30 min at 4 °C. For intracellular staining, splenocytes were stimulated for 4 h with Cell Activation Cocktail (phorbol-12-myristate 13-acetate, ionomycin and Brefeldin A) (Biolegend, San Diego, CA, USA) according to the manufacturer’s protocol. The cells were surface stained with antibody binding cell-specific surface marker and fixed and permeabilized using Fixative Buffer (Biolegend, San Diego, CA, USA) and Intracellular Staining Perm Wash Buffer (Biolegend, San Diego, CA, USA), respectively. Then, incubated with antibodies binding specific intercellular marker. Cell fluorescence was assessed with a FACS AriaII (BD Biosciences, San Jose, CA, USA) and analyzed with FlowJo software (version 10.6.2).

### 4.7. Isolation of Macrophages, DCs and CD4+ T Cells

Spleens from the tumor-bearing Balb/C mice or mice after cryo-thermal therapy were harvested and splenocytes were prepared using GentleMACS™ dissociator (Miltenyi Biotec, Bergisch Gladbach, Germany) and passed through a 70-μm nylon filter. CD68^+^ macrophage isolation by using EasySepTM PE positive selection kit (StemCell Technologies, Vancouver, BC, Canada) and CD68-PE (clone FA-11, Biolegend, San Diego, CA, USA) according to the manufacturer’s instructions. DCs were isolated from splenocytes by using DCs isolation micro-bead kit (EasysepTM CD11c positive selection kit, StemCell Technologies, Vancouver, BC, Canada) according to the manufacturer’s instructions. CD4+ T cells were purified from splenocytes using Easysep mouse CD4^+^ T cell Enrichment Kits (StemCell Technologies, Vancouver, BC, Canada) according to the manufacturer’s instructions. Cells with a purity of >90% were used for experiments.

### 4.8. Histology and Immunohistochemistry Stain

For Hematoxylin and Eosin (H&E) staining, tissues were fixed for 24 h in 10% neutral buffered formalin, dehydrated and embedded in paraffin. Tissue sections (3–5 µm) were stained with H&E. Images were acquired with KFBIO KF-PRO-120 digital pathology slide scanner.

The frozen tissues were sliced into sections of 10 μm each at −20 °C and fixed for 10 min in 4% paraformaldehyde. Tissue sections were staining with Perls’ Iron Stain Kit (Solarbio, Beijing, China) following manufacturer’s instructions. When indicated, Perls’ staining was further enhanced using the DAB-kit (Beyotime, Shanghai, China). For immunohistochemistry, tissue sections were fixed, washed in PBS and treated with H_2_O_2_ to block endogenous peroxidase. Sections were incubated with anti-F4/80 (clone BM8, Biolegend, San Diego, CA, USA, 1:500) at 4 ℃ overnight and were incubated with HRP-labeled Goat Anti-Rat IgG (H + L) (Beyotime, Shanghai, China, 1:100) at 37 °C for 1 h and then stained with diaminobenzidine (DAB) at room temperature for 10 min in the dark, followed by counterstaining with hematoxylin for cell nuclei. Images were acquired with a Leica microscope.

### 4.9. RNA Isolation and Real-Time qPCR

Total RNA was prepared from purified CD68^+^ macrophages, CD11c^+^ DCs, RAW264.7 cells and peritoneal macrophages respectively, using RNAiso Plus Reagent (TaKaRa, Dalian, China). Absorbance at 260/280 nm for mRNA purity at a ratio above 1.9 was achieved for all samples used. cDNA was made using a PrimerScript RT reagent kit with gDNA Eraser (TaKaRa, Dalian, China). Quantitative real-time PCR (RT-PCR) was performed on ABI 7900HT sequence detection system and SDS software (Applied Biosystems, Foster City, CA, USA) using SYBR Premix Ex Taq (TaKaRa, Dalian, China) and samples were amplified in 384-well plates. The primer sequences of mouse genes present in Table S1. Relative expression levels of mRNA for each gene were normalized to GAPDH determined by using the Ct value and assessed using relative quantification (delta–delta Ct method). All experiments were performed in triplicates.

### 4.10. Preparation of Whole Cell Lysates and Western Blot Analysis

CD68^+^ macrophages were lysed in 1×RIPA Lysis Buffer with 1:100 proteinase inhibitor cocktail (Thermo Fisher Scientific, Bremencity, Germany) added before use and incubated on ice for 30 min. Twenty micrograms of protein was resolved by 10% or 12.5% SDS-PAGE and transferred to a polyvinylidene fluoride (PVDF) membrane. Antibody against ferritin was obtained from Abcam and antibody against GAPDH were obtained from Beyotime. Western blot analysis was performed using the primary antibodies and was detected using the appropriate HRP-labeled secondary antibody (EpiZyme, Shanghai, China) and enhanced chemiluminescence (Pierce, Rockford, IL, USA). Each Western blot shown is representative of three separate experiments.

### 4.11. Iron Chelation In Vitro

RAW264.7 cells were cultured in DEME medium (Hyclone, Logan, UT, USA) supplemented with 10% fetal bovine serum (FBS) and penicillin-streptomycin (Hyclone, Logan, UT, USA). To prepare peritoneal macrophages, Balb/C mice were injected intraperitoneally with 2 mL of sterile 3% Thioglycollate Broth (Sigma-Aldrich, St Louis, MO, USA) per mouse. On day 3 after injection, macrophages were harvested from the peritoneal cavity by lavaging with sterile phosphate-buffered saline. The macrophages were centrifuged at 400× *g* for 10 min, resuspended in RPMI 1640 medium (Gibco, California, CA, USA) with 10% FBS and penicillin-streptomycin (Hyclone, Logan, UT, USA) and distributed into the wells of 24-well plates. RAW264.7 cells, peritoneal macrophages, splenic macrophages (isolated from tumor-bearing group) and splenic CD11c^+^ DCs (isolated from tumor-bearing group) were treated with 2% tumor interstitial fluid and 200 μM DFO for 24 h and then analyzed by flow cytometry, western blot and Real-time qPCR. In some experiments, cells were pretreated with U0126 (Cell Signaling Technology, Massachusetts, MA, USA) for 30 min to inhibit the phosphorylation of ERK.

### 4.12. In Vitro Cell Co-Culture

For understanding the role of iron-induced macrophages after cryo-thermal therapy in DCs phenotypic maturation and activation, the isolated CD68^+^ macrophages from tumor-bearing mice, mice 24 h after receiving cryo-thermal therapy and mice 24 h after receiving cryo-thermal therapy combined with DFO intratumoral injection were cocultured with CD11c^+^ DCs from the tumor-bearing mice at a ratio of 1:1 for 24 h and the cocultured DCs were analyzed by flow cytometry. For understanding the role of iron-induced macrophages and DCs in the differentiation and cytotoxicity of CD4^+^ T cell, macrophages and DCs from each group of mice were cocultured with CD4+ T cell from the tumor-bearing mice at a ratio of 1:5 for 24 h and the cocultured CD4^+^ T cell were detected by flow cytometry.

### 4.13. Statistical Analysis

The results were expressed as the mean ± standard deviation (SD). Statistical analyses were conducted with Student’s *t*-test. A *p*-value of <0.05 was regarded as statistically significant.

## Data Availability

The data presented in this study are available on request from the corresponding author.

## Supplementary Materials

The following are available online at https://www.mdpi.com/article/10.3390/ijms22137010/s1.

## References

[1] Qin H.W., Holdbrooks A.T., Liu Y.D., Reynolds S.L., Yanagisawa L.L., Benveniste E.N. (2012). SOCS3 Deficiency Promotes M1 Macrophage Polarization and Inflammation. J. Immunol..

[2] Mantovani A., Locati M. (2009). Orchestration of macrophage polarization. Blood.

[3] Mantovani A., Allavena P. (2015). The interaction of anticancer therapies with tumor-associated macrophages. J. Exp. Med..

[4] Noy R., Pollard J.W. (2014). Tumor-Associated Macrophages: From Mechanisms to Therapy (vol 41, pg 49, 2014). Immunity.

[5] Engblom C., Pfirschke C., Pittet M.J. (2016). The role of myeloid cells in cancer therapies. Nat. Rev. Cancer.

[6] Brown J.M., Recht L., Strober S. (2017). The Promise of Targeting Macrophages in Cancer Therapy. Clin. Cancer Res..

[7] Gutierrez-Gonzalez A., Martinez-Moreno M., Samaniego R., Arellano-Sanchez N., Salinas-Munoz L., Relloso M., Valeri A., Martinez-Lopez J., Corbi A.L., Hidalgo A. (2016). Evaluation of the potential therapeutic benefits of macrophage reprogramming in multiple myeloma. Blood.

[8] Zhu J., Zhang Y., Zhang A., He K., Liu P., Xu L.X. (2016). Cryo-thermal therapy elicits potent anti-tumor immunity by inducing extracellular Hsp70-dependent MDSC differentiation. Sci. Rep..

[9] He K., Liu P., Xu L.X. (2017). The cryo-thermal therapy eradicated melanoma in mice by eliciting CD4(+) T-cell-mediated antitumor memory immune response. Cell Death Dis..

[10] Xue T., Liu P., Zhou Y., Liu K., Yang L., Moritz R.L., Yan W., Xu L.X. (2016). Interleukin-6 Induced “Acute” Phenotypic Microenvironment Promotes Th1 Anti-Tumor Immunity in Cryo-Thermal Therapy Revealed By Shotgun and Parallel Reaction Monitoring Proteomics. Theranostics.

[11] Cai Z.H., Song M.Y., Zhang A.L., Sun J.Q., Xu L.X.M. (2013). Numerical Simulation of a New Probe for the Alternate Cooling and Heating of a Subcutaneous Mouse Tumor Model. Numer. Heat Transf. Part A Appl..

[12] Liu P., Ren X.M., Xu L.X. Alternate Cooling and Heating Thermal Physical Treatment: An Effective Strategy against Mdscs in 4t1 Mouse Mammary Carcinoma. Proceedings of the ASME 2012 Summer Bioengineering Conference, Parts A and B.

[13] Sun J.Q., Xu C.C., Wei G.H., Sun X.G., Liu P., Zhang A.L., Xu L.X. (2009). Tumor Treatment System with Alternate Cooling and Heating—Preliminary Results in an Animal Model. IFMBE Proc..

[14] Sun J.Q., Zhang A.L., Xu L.X. (2008). Evaluation of alternate cooling and heating for tumor treatment. Int. J. Heat Mass Transf..

[15] Liu P., Jia S.G., Lou Y., He K., Xu L.S.X. (2019). Cryo-thermal therapy inducing MI macrophage polarization created CXCL10 and IL-6-rich pro-inflammatory environment for CD4(+) T cell-mediated anti-tumor immunity. Int. J. Hyperth..

[16] He K., Jia S.G., Lou Y., Liu P., Xu L.X. (2019). Cryo-thermal therapy induces macrophage polarization for durable anti-tumor immunity. Cell Death Dis..

[17] Zhu J., Lou Y., Liu P., Xu L.X. (2020). Tumor-related HSP70 released after cryo-thermal therapy targeted innate immune initiation in the antitumor immune response. Int. J. Hyperth..

[18] Soares M.P., Hamza I. (2016). Macrophages and Iron Metabolism. Immunity.

[19] Korolnek T., Hamza I. (2015). Macrophages and iron trafficking at the birth and death of red cells. Blood.

[20] Cairo G., Recalcati S., Mantovani A., Locati M. (2011). Iron trafficking and metabolism in macrophages: Contribution to the polarized phenotype. Trends Immunol..

[21] Hoeft K., Bloch D.B., Graw J.A., Malhotra R., Ichinose F., Bagchi A. (2017). Iron Loading Exaggerates the Inflammatory Response to the Toll-like Receptor 4 Ligand Lipopolysaccharide by Altering Mitochondrial Homeostasis. Anesthesiology.

[22] Kroner A., Greenhalgh A.D., Zarruk J.G., Passos Dos Santos R., Gaestel M., David S. (2014). TNF and increased intracellular iron alter macrophage polarization to a detrimental M1 phenotype in the injured spinal cord. Neuron.

[23] Sindrilaru A., Peters T., Wieschalka S., Baican C., Baican A., Peter H., Hainzl A., Schatz S., Qi Y., Schlecht A. (2011). An unrestrained proinflammatory M1 macrophage population induced by iron impairs wound healing in humans and mice. J. Clin. Investig..

[24] Zanganeh S., Hutter G., Spitler R., Lenkov O., Mahmoudi M., Shaw A., Pajarinen J.S., Nejadnik H., Goodman S., Moseley M. (2016). Iron oxide nanoparticles inhibit tumour growth by inducing pro-inflammatory macrophage polarization in tumour tissues. Nat. Nanotechnol..

[25] Zhou Y., Que K.T., Zhang Z., Yi Z.J., Zhao P.X., You Y., Gong J.P., Liu Z.J. (2018). Iron overloaded polarizes macrophage to proinflammation phenotype through ROS/acetyl-p53 pathway. Cancer Med..

[26] Thielmann C.M., da Silva M.C., Muley T., Meister M., Herpel E., Muckenthaler M.U. (2019). Iron accumulation in tumor-associated macrophages marks an improved overall survival in patients with lung adenocarcinoma. Sci. Rep..

[27] Elliott R.L., Elliott M.C., Wang F., Head J.F. (1993). Breast carcinoma and the role of iron metabolism. A cytochemical, tissue culture, and ultrastructural study. Ann. N. Y. Acad. Sci..

[28] Güner G., Kirkali G., Yenisey C., Töre I.R. (1992). Cytosol and serum ferritin in breast carcinoma. Cancer Lett..

[29] Weinstein R.E., Bond B.H., Silberberg B.K. (1982). Tissue ferritin concentration in carcinoma of the breast. Cancer.

[30] Torti S.V., Torti F.M. (2013). Iron and cancer: More ore to be mined. Nat. Rev. Cancer.

[31] Duan X., He K., Li J., Cheng M., Song H., Liu J., Liu P. (2018). Tumor associated macrophages deliver iron to tumor cells via Lcn2. Int. J. Physiol. Pathophysiol. Pharm..

[32] Zhu F., Qin B.J., Feng W.Y., Wang H.J., Huang S.S., Lv Y.S., Chen Y. (2013). Reducing Poisson noise and baseline drift in X-ray spectral images with bootstrap Poisson regression and robust nonparametric regression. Phys. Med. Biol..

[33] Shen Y.Y., Liu P., Zhang A.L., Xu L.X. (2008). Study on tumor microvasculature damage induced by alternate cooling and heating. Ann. Biomed. Eng..

[34] Nairz M., Theurl I., Swirski F.K., Weiss G. (2017). “Pumping iron”-how macrophages handle iron at the systemic, microenvironmental, and cellular levels. Pflug. Arch. Eur. J. Physiol..

[35] Costa da Silva M., Breckwoldt M.O., Vinchi F., Correia M.P., Stojanovic A., Thielmann C.M., Meister M., Muley T., Warth A., Platten M. (2017). Iron Induces Anti-tumor Activity in Tumor-Associated Macrophages. Front. Immunol..

[36] Arosio P., Elia L., Poli M. (2017). Ferritin, Cellular Iron Storage and Regulation. LUBMB Life.

[37] La A., Nguyen T., Tran K., Sauble E., Tu D., Gonzalez A., Kidane T.Z., Soriano C., Morgan J., Doan M. (2018). Mobilization of iron from ferritin: New steps and details. Met. Integr. Biomet. Sci..

[38] Ma X., Yan W., Zheng H., Du Q., Zhang L., Ban Y., Li N., Wei F. (2015). Regulation of IL-10 and IL-12 production and function in macrophages and dendritic cells. F1000Research.

[39] Yiu S.P.T., Hui K.F., Choi C.K., Kao R.Y.T., Ma C.W., Yang D., Chiang A.K.S. (2018). Intracellular Iron Chelation by a Novel Compound, C7, Reactivates Epstein-Barr Virus (EBV) Lytic Cycle via the ERK-Autophagy Axis in EBV-Positive Epithelial Cancers. Cancers.

[40] Han X., Huang S., Xue P., Fu J., Liu L., Zhang C., Yang L., Xia L., Sun L., Huang S.K. (2019). LncRNA PTPRE-AS1 modulates M2 macrophage activation and inflammatory diseases by epigenetic promotion of PTPRE. Sci. Adv..

[41] Mu X.M., Shi W., Xu Y., Xu C., Zhao T., Geng B., Yang J., Pan J.S., Hu S., Zhang C. (2018). Tumor-derived lactate induces M2 macrophage polarization via the activation of the ERK/STAT3 signaling pathway in breast cancer. Cell Cycle.

[42] Kang H., Zhang J., Wang B., Liu M., Zhao J., Yang M., Li Y. (2017). Puerarin inhibits M2 polarization and metastasis of tumor-associated macrophages from NSCLC xenograft model via inactivating MEK/ERK 1/2 pathway. Int. J. Oncol..

[43] Goodridge H.S., Harnett W., Liew F.Y., Harnett M.M. (2003). Differential regulation of interleukin-12 p40 and p35 induction via Erk mitogen-activated protein kinase-dependent and -independent mechanisms and the implications for bioactive IL-12 and IL-23 responses. Immunology.

[44] Liu K., He K., Xue T., Liu P., Xu L.X. (2018). The cryo-thermal therapy-induced IL-6-rich acute pro-inflammatory response promoted DCs phenotypic maturation as the prerequisite to CD4(+) T cell differentiation. Int. J. Hyperth..

[45] Asadzadeh Z., Mohammadi H., Safarzadeh E., Hemmatzadeh M., Mahdian-Shakib A., Jadidi-Niaragh F., Azizi G., Baradaran B. (2017). The paradox of Th17 cell functions in tumor immunity. Cell Immunol.

[46] Jia S., Li W., Liu P., Xu L.X. (2019). A role of eosinophils in mediating the anti-tumour effect of cryo-thermal treatment. Sci. Rep..

[47] Peng P., Hu H.M., Liu P., Xu L.X. (2020). Neoantigen-specific CD4(+)T-cell response is critical for the therapeutic efficacy of cryo-thermal therapy. J. Immunother. Cancer.

[48] Vinchi F., Costa da Silva M., Ingoglia G., Petrillo S., Brinkman N., Zuercher A., Cerwenka A., Tolosano E., Muckenthaler M.U. (2016). Hemopexin therapy reverts heme-induced proinflammatory phenotypic switching of macrophages in a mouse model of sickle cell disease. Blood.

[49] Paller M.S., Hedlund B.E. (1994). Extracellular iron chelators protect kidney cells from hypoxia/reoxygenation. Free Radic. Biol. Med..

[50] Wang S., Liu C., Pan S., Miao Q., Xue J., Xun J., Zhang Y., Gao Y., Duan X., Fan Y. (2015). Deferoxamine attenuates lipopolysaccharide-induced inflammatory responses and protects against endotoxic shock in mice. Biochem. Biophys. Res. Commun..

[51] Mertens C., Akam E.A., Rehwald C., Brune B., Tomat E., Jung M. (2016). Intracellular Iron Chelation Modulates the Macrophage Iron Phenotype with Consequences on Tumor Progression. PLoS ONE.

[52] Li T., Wu B., Yang T., Zhang L., Jin K. (2020). The outstanding antitumor capacity of CD4(+) T helper lymphocytes. Biochim. Biophys. Acta Rev. Cancer.

[53] Takeuchi A., Saito T. (2017). CD4 CTL, a Cytotoxic Subset of CD4(+) T Cells, Their Differentiation and Function. Front. Immunol..

[54] Hong S.Y., Qian J.F., Yang J., Li H.Y., Kwak L.W., Yi Q. (2008). Roles of Idiotype-Specific T Cells in Myeloma Cell Growth and Survival: Th1 and CTL Cells Are Tumoricidal while Th2 Cells Promote Tumor Growth. Cancer Res..

[55] Salazar Y., Zheng X., Brunn D., Raifer H., Picard F., Zhang Y.J., Winter H., Guenther S., Weigert A., Weigmann B. (2020). Microenvironmental Th9 and Th17 lymphocytes induce metastatic spreading in lung cancer. J. Clin. Investig..

[56] Benevides L., da Fonseca D.M., Donate P.B., Tiezzi D.G., De Carvalho D.D., de Andrade J.M., Martins G.A., Silva J.S. (2015). IL17 Promotes Mammary Tumor Progression by Changing the Behavior of Tumor Cells and Eliciting Tumorigenic Neutrophils Recruitment. Cancer Res..

[57] Shen L.S., Zhou Y.X., He H.F., Chen W.Z., Lenahan C., Li X.Y., Deng Y.C., Shao A.W., Huang J. (2021). Crosstalk between Macrophages, T Cells, and Iron Metabolism in Tumor Microenvironment. Oxid. Med. Cell. Longev..

