# Supplementary material for: Iron Released after Cryo-Thermal Therapy Induced M1 Macrophage Polarization, Promoting the Differentiation of CD4+ T Cells into CTLs
Source: Int J Mol Sci. 2021 Jun 29;22(13):7010. doi: 10.3390/ijms22137010 (PMC8268875; doi:10.3390/ijms22137010)
Supplement: Supplementary file 1 [file ijms-22-07010-s001.zip › ijms-1245077-supplementary.pdf]

Supplementary Material

Supplementary Figures

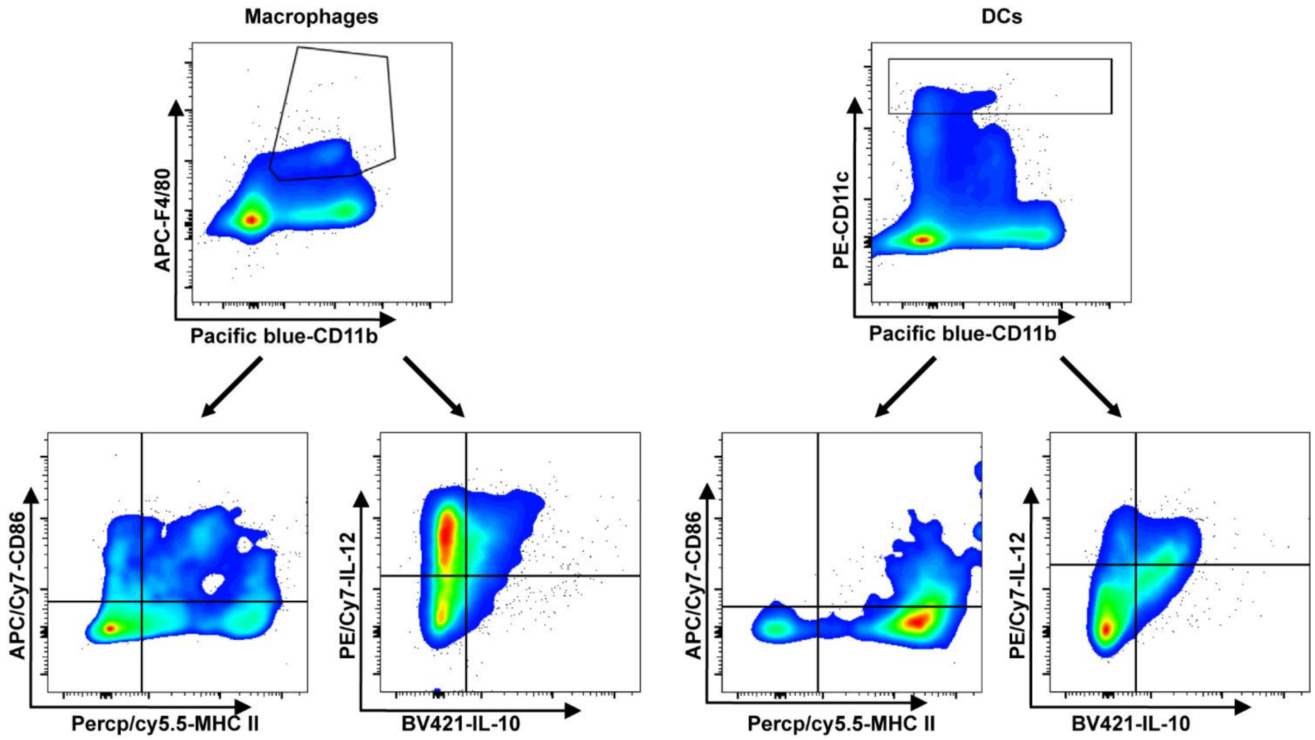

Figure S1. Gating strategy of flow cytometry for the M1 macrophages, mature DCs and intracellular staining of IL-12 and IL-10.

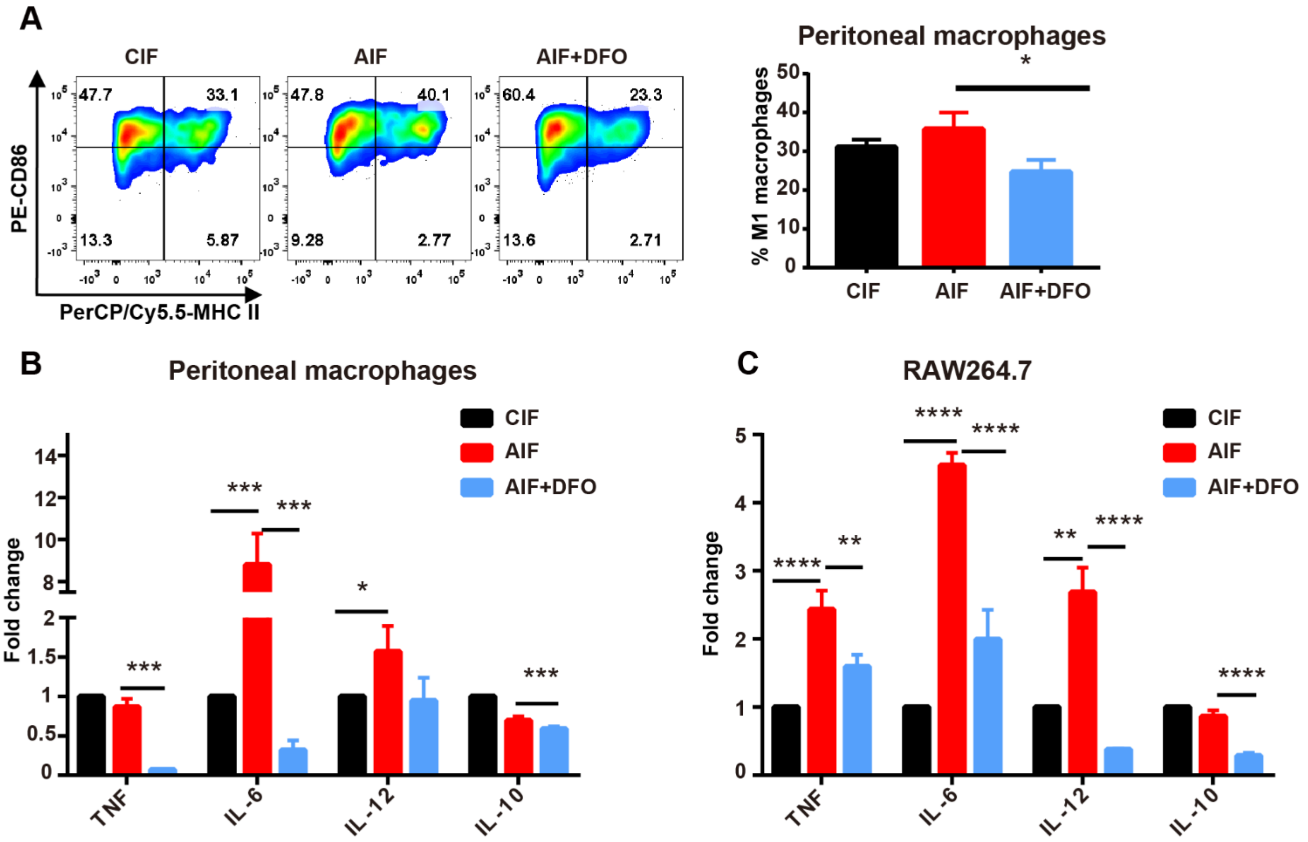

Figure S2. Tumor interstitial fluid harversted from cryo-thermal therapy treated mice (AIF) promoted the M1 polarization of peritoneal macrophages and RAW264.7 cells. Cells were cultured with tumor interstitial fluid from tumor-bearing mice (CIF) or treated mice (AIF). When indicated, DFO (200 μM) was added. After 24 h of cultivation, (A) the percentage of M1 macrophages ($CD86^{+}MHCII^{+}$) determined by flow cytometer. (B) The expression levels of *TNF*, *IL-6*, *IL-12* and *IL-10* in peritoneal macrophages and RAW264.7 cells were determined by RT-qPCR. Data were show as mean±SD, and the student's t-test is used for statistical analysis. *p<0.05, **p<0.01, ***p<0.001, ****p<0.0001.

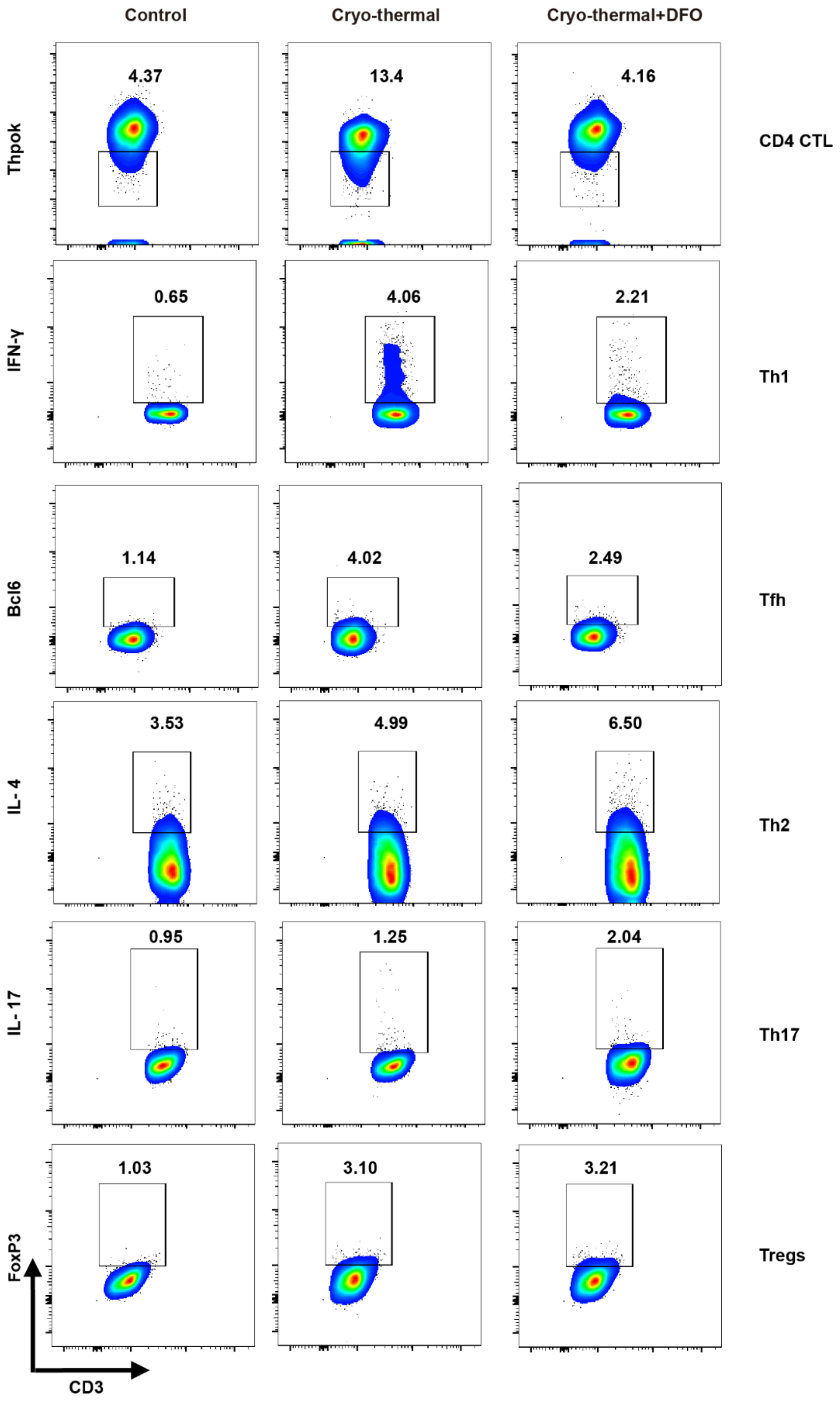

Figure S3. Represented flow cytometry graph of CD4 CTL (Thpok$^-$), Th1(IFN-γ^+), Tfh (Bcl6$^+$), Th2(IL-4$^+$), Th17(IL-17$^+$) and Treg (Foxp3$^+$) subsets in CD4$^+$ T cells.

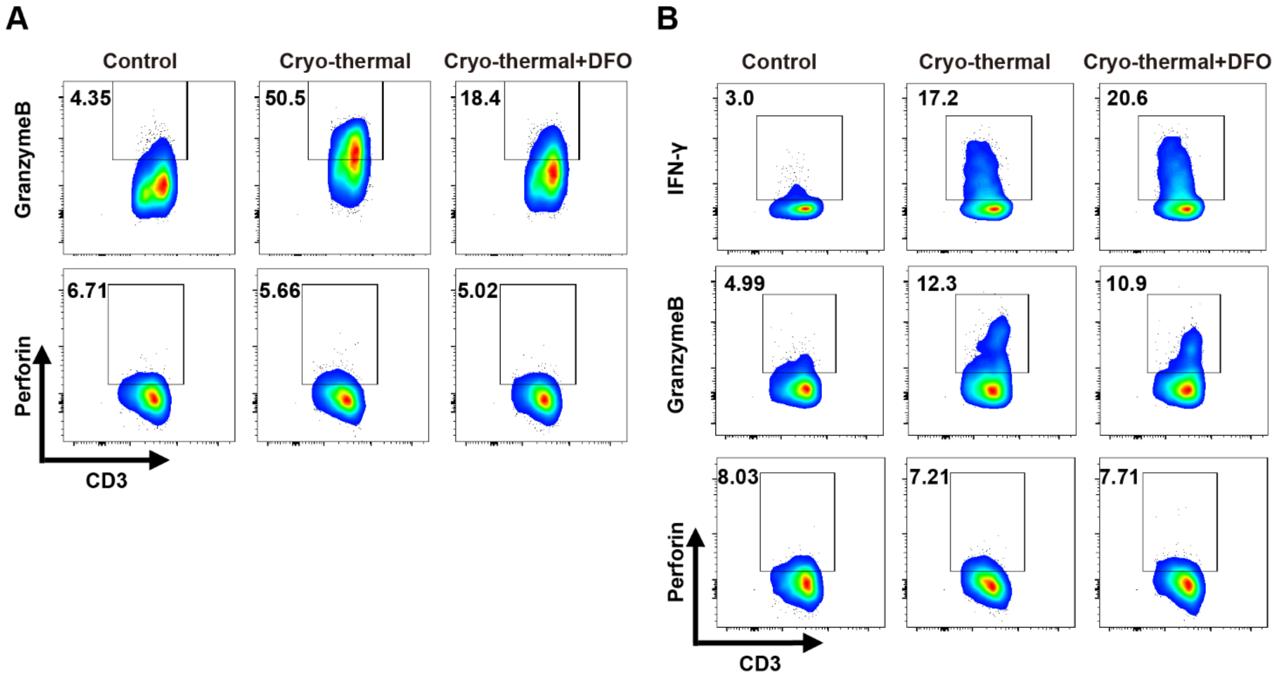

Figure S4. Represented flow cytometry graph for granzyme B$^+$ and perforin$^+$ cells in CD4$^+$ T cells (A) and IFN-γ$^+$ granzyme B$^+$ and perforin$^+$ cells in CD8$^+$ T cells (B).

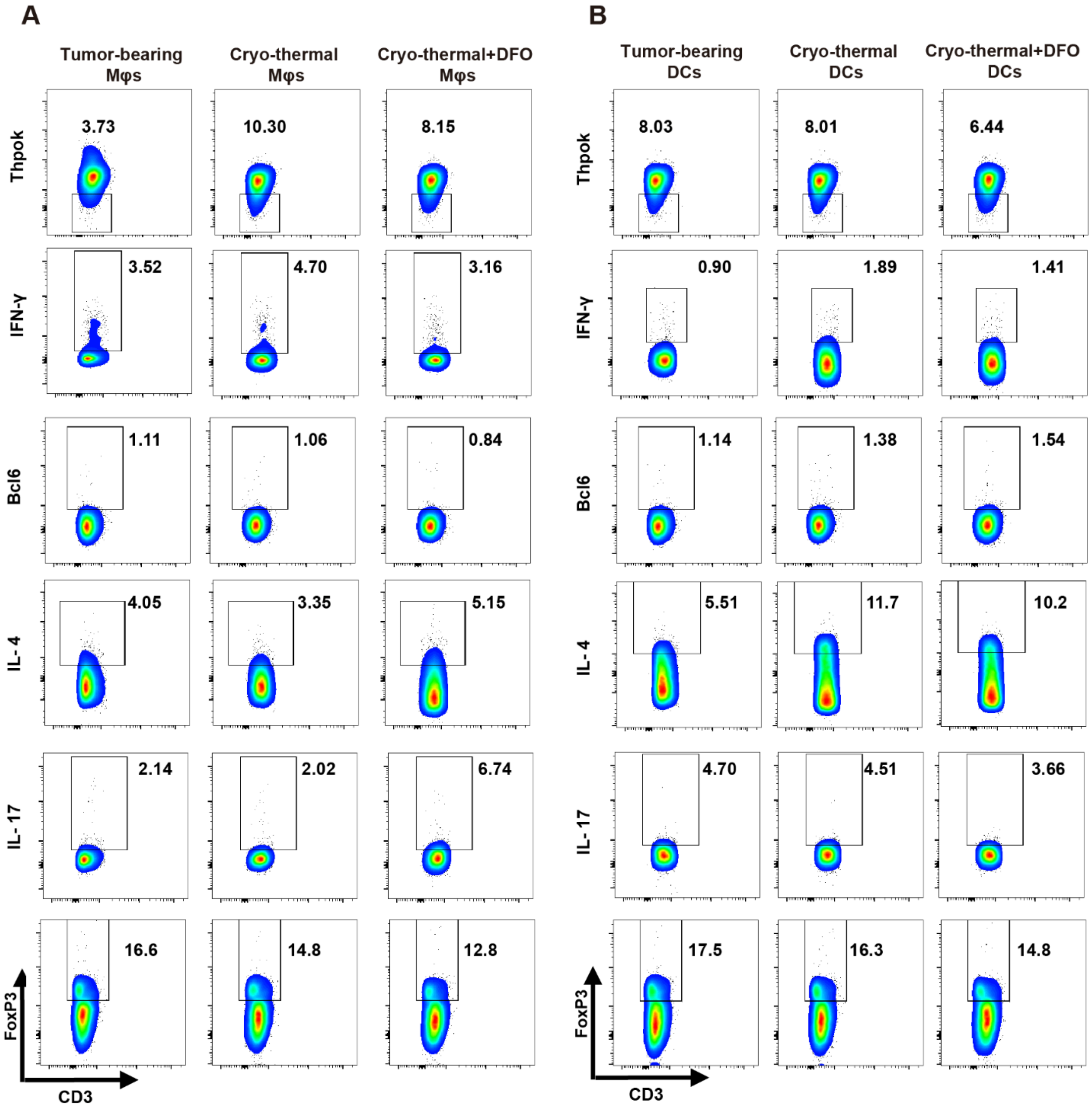

Figure S5. Represented flow cytometry graph of CD4 CTL (Thpok$^-$), Th1(IFN-γ^+), Tfh (Bcl6$^+$), Th2(IL-4$^+$), Th17(IL-17$^+$) and Treg (Foxp3$^+$) subsets in CD4$^+$ T cells after cocultured with macrophages (A) and DCs (B) sorted from tumor-bearing mice, cryo-thermal group and DFO treated cryo-thermal group mice.

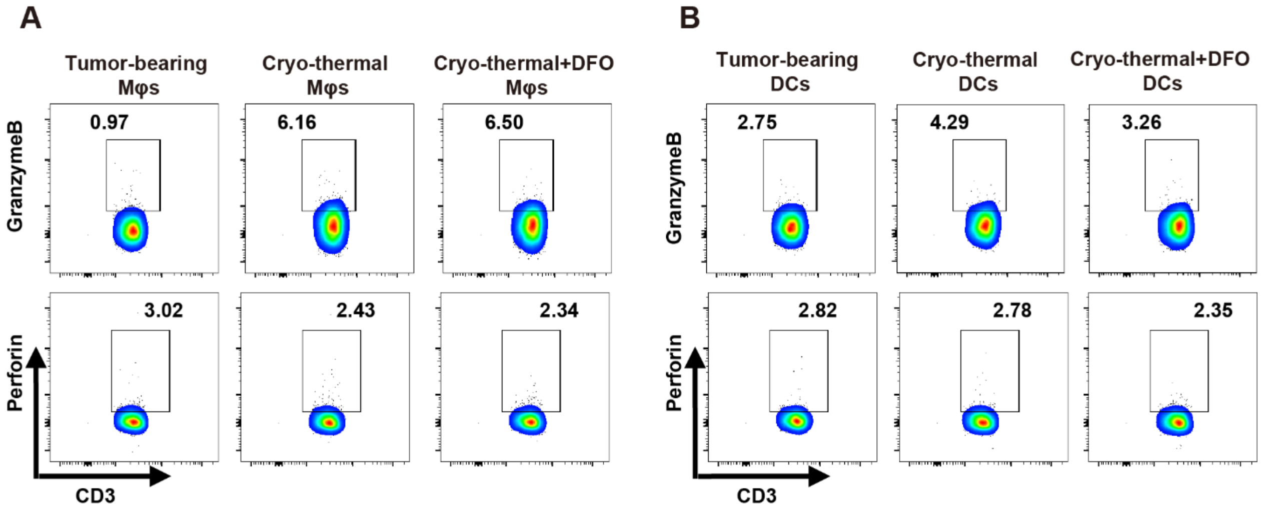

Figure S6. Represented flow cytometry graph for granzyme B^{+} and perforin^{+} cells in CD4^{+} T cells after cocultured with macrophages (A) and DCs (B) sorted from tumor-bearing mice, cryo-thermal group and DFO treated cryo-thermal group mice.

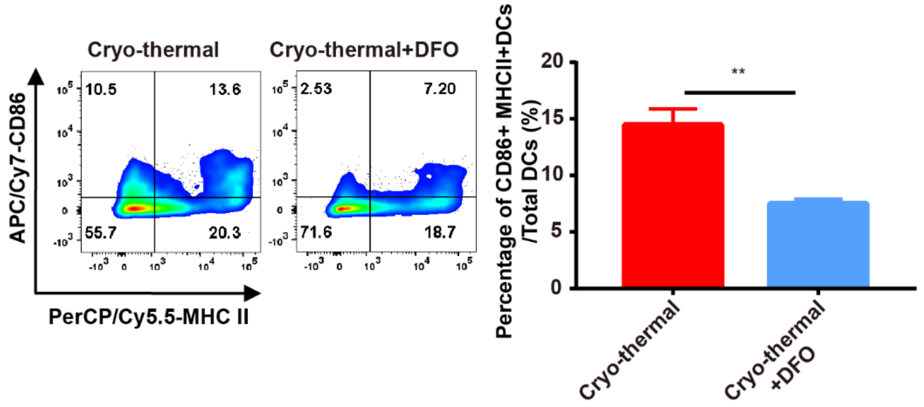

Figure S7. Iron-induced M1 macrophages promoted the maturation of DCs. Splenic macrophages(CD68$^+$) sorted from mice receiving cryo-thermal therapy or cryo-thermal therapy combined with DFO treatment and co-cultured with tumor-bearing DCs at a ratio of 1:1 for 24 h. The percentage of mature DCs (CD86$^+$ MHC II$^+$) was detected by flow cytometry. Data were show as mean±SD, and the student's t-test is used for statistical analysis. **p<0.01.

Supplementary Table

Table S1. Primer sequences of various genes in this study.

Name	Primer Sequence (5´-3´)
FtH-forward	CAAGTGCGCCAGAACTACCA
FtH-reverse	ACAGATAGACGTAGGAGGCATAC
TNF-forward	TTCTGTCTACTGAACTTCGGGGTGATCGGTCC
TNF-reverse	GTATGAGATAGCAAATCGGCTGACGGTGTGGG
IL-6-forward	GACAAAGCCAGAGTCCTTCAGAGAGATACAG
IL-6-reverse	TTGGATGGTCTTGGTCCTTAGCCAC
IL-12-forward	TGGTTTGCCATCGTTTTGCTG
IL-12-reverse	ACAGGTGAGGTTCACTGTTTCT
IL-10-forward	GCTCTTACTGACTGGCATGAG
IL-10-reverse	CGCAGCTCTAGGAGCATGTG
CXCL10-forward	CCAAGTGCTGCCGTCATTTTC
CXCL10-reverse	GGCTCGCAGGGATGATTTCAA
GAPDH-forward	AGGTCGGTGTGAACGGATTTG
GAPDH- reverse	GGGGTCGTTGATGGCAACA
